# Transcriptome-wide survey of pseudorabies virus using next- and third-generation sequencing platforms

**DOI:** 10.1038/sdata.2018.119

**Published:** 2018-06-19

**Authors:** Dóra Tombácz, Donald Sharon, Attila Szűcs, Norbert Moldován, Michael Snyder, Zsolt Boldogkői

**Affiliations:** 1 Department of Medical Biology, Faculty of Medicine, University of Szeged, Szeged 6720, Hungary; 2 Department of Genetics, School of Medicine, Stanford University, Stanford, California 94305, USA

**Keywords:** Transcriptomics, Herpes virus, RNA sequencing, Gene expression

## Abstract

Pseudorabies virus (PRV) is an alphaherpesvirus of swine. PRV has a large double-stranded DNA genome and, as the latest investigations have revealed, a very complex transcriptome. Here, we present a large RNA-Seq dataset, derived from both short- and long-read sequencing. The dataset contains 1.3 million 100 bp paired-end reads that were obtained from the Illumina random-primed libraries, as well as 10 million 50 bp single-end reads generated by the Illumina polyA-seq. The Pacific Biosciences RSII non-amplified method yielded 57,021 reads of inserts (ROIs) aligned to the viral genome, the amplified method resulted in 158,396 PRV-specific ROIs, while we obtained 12,555 ROIs using the Sequel platform. The Oxford Nanopore’s MinION device generated 44,006 reads using their regular cDNA-sequencing method, whereas 29,832 and 120,394 reads were produced by using the direct RNA-sequencing and the Cap-selection protocols, respectively. The raw reads were aligned to the PRV reference genome (KJ717942.1). Our provided dataset can be used to compare different sequencing approaches, library preparation methods, as well as for validation and testing bioinformatic pipelines.

## Background & Summary

Pseudorabies virus (PRV) is a causative agent of Aujeszky’s disease (AD)^1^ in pigs. PRV has a double-stranded DNA genome with a size of approximately 143 kbp. PRV is often employed in laboratories to study the molecular pathomechanism of the herpesviruses^2^. It is also a suitable tool as concerns gene and tumour therapy^3^, as well as for mapping of neuronal circuits^4–8^. This virus has also been used as live vaccines against AD^9–11^.

**Figure 1:**
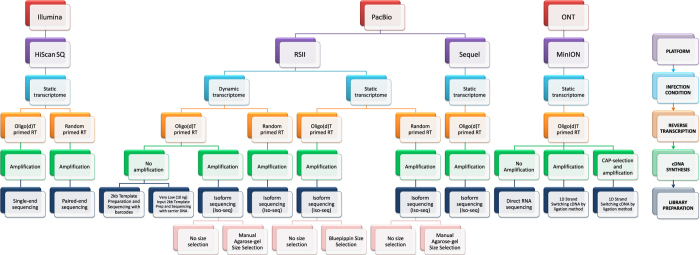
Data flow diagram shows the detailed overview of the study design.

Here, we provide a large dataset derived from RNA-Seq experiments including different next-generation sequencing (NGS) – and third-generation sequencing (3^rd^GS) techniques (Fig. 1). Our aim with this study was to provide a dataset that can be used for comparison of the different sequencing platforms and library preparation methods using PRV as a model organism. In addition, these data are also applicable for identifying novel coding and non-coding transcripts, transcript isoforms, splice variants of PRV, and for defining full-length transcripts by using a combination of sequencing platforms.

One of the most popular NGS platforms, the Illumina HiScanSQ was used to generate high quality short-reads and extremely high coverage throughout the entire PRV genome. Random-primed cDNA library was prepared from viral RNAs. Paired-end RNA sequencing was carried out to characterize novel splice isoforms, as well as to obtain general information on the transcription activity of PRV^12^. PolyA-sequencing was used to determine the 3′-ends of RNA molecules. With this technique, we were able to detect alternative polyadenylation events in the PRV transcripts. Both libraries were run on a single flow cell resulting in 1.3 million 100 bp paired-end reads from the random hexamer-primed libraries, and 10 million 50 bp single-end reads from the poly(A)-enriched RNA-Seq, aligning to the viral reference^13^ (KJ717942.1).

Although the error rate of 3^rd^GS techniques is higher than those of NGS’s^14^, they are able to identify novel full-length transcripts^15–17^ and are therefore more applicable for global transcriptome profiling and RNA isoform detection compared to short-read techniques.

**Figure 2:**
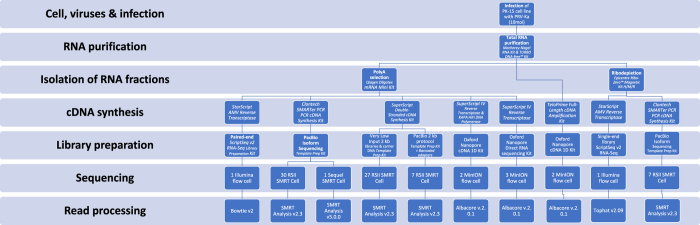
Data flow diagram shows the detailed overview of the wet lab experiments and bioinformatics pipelines.

The Real-Time Sequencer II (RSII) and the Sequel 3^rd^GS platforms from Pacific Biosciences (PacBio) and the Oxford Nanopore Technologies (ONT) MinION 3^rd^GS device were used to characterize the static^18,19^ and dynamic^20^ PRV transcriptome. These sequencing techniques, with the library preparation methods [e.g. non-amplified SMRT method and amplified, Iso-seq protocol from the PacBio; full-length cDNA-sequencing, direct RNA-sequencing, and cDNA-sequencing on 5′Cap-selected samples from ONT, (Fig. 1,2)] used in these studies made it possible to identify several hundreds of novel transcript isoforms (including 3′- and 5′ UTR variants, and splice isoforms), as well as dozens of protein-coding and non-coding RNAs and numerous complex transcripts of PRV.

Seventy-one SMRT Cells were run on RSII system. P5-C3 chemistry and 180-minute data collection mode was used for the non-amplified samples, while P6-C4 enzymes were applied and 240 or 360 min movies were recorded for the amplified samples. cDNAs were sequenced on a single Sequel SMRT Cell with P6-C4 reagents; 10 h run-time was applied. Altogether seven MinION flow cells were used for the different ONT approaches.

The raw sequencing reads were mapped to the above-mentioned reference genome. Sequencing on the RSII platform resulted in 215,417 reads of inserts (ROIs), while the utilized nanopore sequencing methods generated altogether 194,232 PRV specific reads (Table 1). The average read lengths aligning to the PRV genome were 1,326 bp for PacBio RSII, 1,763 bp for the Sequel and 827 bp for ONT. It should be noted that the library preparation and size-selection methods resulted in different samples in length (Table 2).

This dataset can help explore the advantages and disadvantages associated with each sequencing method used in this work. This approach can be used for the analysis of multiple features of the sequencing platforms, including read length, base-calling error rate, coverage and mappability. The application of the various sequencing techniques can be evaluated by the analysis of the identified transcript isoforms, and the quantification of the transcriptome comparing the performance of Illumina, PacBio and ONT. This dataset is also useful for the analysis of the transcriptome complexity of PRV. Our data include a sub-dataset which can be used for the transcriptome analysis of PRV during an infection period including six different time-points.

Here we provide a detailed overview of the library preparation techniques and a description of the data (Table 3, Figs. 1 and 2).

## Methods

Schematic overviews of the methodological workflow are shown in the flowchart of Figs. 1 and 2. The applied reagents and utilized approaches are listed in Table 4.

### Cells, viruses and infection conditions

Immortalized porcine kidney-15 (PK-15; ATCC® CCL-33™) cells were used for the propagation of pseudorabies virus strain Kaplan (PRV-Ka) at 37 °C and 5% CO_2_ in Dulbecco’s modified Eagle medium (DMEM, Gibco Invitrogen) supplemented with 5% foetal bovine serum (FBS; Gibco Invitrogen). The virus stock was originally obtained from the Kaplan Lab (Department of Microbiology, Vanderbilt University School of Medicine, Nashville, Tennessee)^21^, but Vanderbilt University received it from Dr. Richard F. Haff in a suspension of infected mouse brain^22^. Gentamycin (80 μg/ml) was also added to the cell culture medium. The virus stock was prepared as follows: the medium was removed from the rapidly-growing semi-confluent PK-15 cells then it was infected with the Kaplan strain of PRV (a multiplicity of infection of 0.1 plaque-forming unit (pfu)/cell). Infected cells were incubated until complete cytopathic effect was observed. Samples were taken through three times freeze-thaw cycles, followed by centrifugation at 10,000 g for 15 min. The titre of the virus stock was determined in PK-15 cells. For all experiments, cells were infected with a high MOI (10 pfu/cell) and incubated for 1 h, followed by removal of the virus suspension and washing of the cells with phosphate-buffered saline (PBS). The number of cells in a culture flask was 5 × 10^6^. After the addition of new medium to the cells, they were incubated for 1, 2, 4, 6, 8, 10, 12, 14, 16, 18, 20, 22 or 24 h pi and they both were mixed for Illumina sequencing (Table 3). One, 2, 4, 6, 8 and 12 h of incubation were used for the non-amplified PacBio sequencing, while the 1, 2, 4, 6 and 8 h pi samples were utilized for the PacBio amplified, Iso-Seq protocol. Samples from different time points were individually sequenced on the RSII, but they were also mixed for PacBio sequencing (Table 3). The incubation time was 1, 2, 3, 4, 5, 6, 7, 8, 9, 12, 18 and 24 h and a mixture from them was used for all types of ONT sequencing (Table 3).

### RNA purification

**Isolation of total RNAs** The NucleoSpin^®^ RNA II kit (Macherey-Nagel) was used to isolate RNA from samples for Illumina sequencing, while the new version, the NucleoSpin^®^ RNA kit (Macherey-Nagel) was used for all the other samples, as was described earlier^12,18,20^. Briefly, cells were collected by centrifugation and lysed by incubation in a solution containing large amounts of chaotropic ions. This buffer inactivates the RNase. Nucleic acid molecules bind to the silica membrane. All samples were handled with DNase I solution (provided by the kit) to remove residual DNA contaminations. Total RNAs were eluted from the membrane in RNase-free water. To eliminate the potential remaining DNA contamination, samples were treated by Ambion® TURBO DNA-free™ Kit. The final concentrations of the RNA samples were determined by Qubit®. 2.0 Fluorometer using Qubit RNA BR Assay Kit (Life Technologies). RNA quality was assessed with the Agilent Bioanalyzer 2100 and RIN scores above 9.6 were used for cDNA production. RNA samples were stored at −80 °C until further use. Samples were made as follows: The Illumina oligodT- and random-primed sequencing reactions were carried out from the same RNA mixture. The libraries for the kinetic analysis and for the mixed sequencing using PacBio RSII (non-amplified method) were all prepared from different cell culture flasks (containing 5×10^6^ cells/flask), but the same virus stock was used for these infections. For the amplified RSII sequencing, samples were prepared from separate flasks (each containing 5×10^6^ cells and infected with the same virus stock). The Sequel and the various MinION libraries have been prepared using the same RNA mixture.

**Ribosomal RNA depletion** For the Illumina sequencing and for the PacBio random-primed sequencing, the total RNA samples were depleted from rRNA using the Epicentre Ribo-Zero™ Magnetic Kit H/M/R (Illumina).

**Selection of PolyA(+) RNA** For the PacBio and MinION polyA sequencing, the polyA(+) fraction of the RNA samples were isolated using the Qiagen Oligotex mRNA Mini Kit, following the “Spin Columns” protocol.

PolyA purified and rRNA depleted RNA samples were quantified through use of the Qubit RNA HS Assay Kit (Life Technologies) and then subjected to cDNA synthesis according to the downstream applications.

### cDNA synthesis, library preparation and sequencing Illumina sequencing

Total RNA was purified from PK-15 cells in various stages of PRV infection from 1 to 24 h pi and then, the samples were mixed together to uncover an extensive variety of viral transcripts. Libraries were prepared from ribo-depleted samples using the ScriptSeq v2 RNA-Seq Library Preparation Kit (Epicentre/Illumina) according to the manufacturer’s recommendations. The kit uses a random-primed (random hexamer with tagging sequence) cDNA synthesis reaction; this “original” protocol was used to construct a paired-end library, but for PolyA sequencing (PA-seq), a single-end library was prepared through the use of custom anchored adaptor-primer oligonucleotides with an oligo(VN)T20 primer sequence (Table 5). Briefly, the rRNA-depleted RNA samples were mixed with the primer (random or oligo(d)T) and the RNA Fragmentation Solution (part of the ScriptSeq Kit), and the mixtures were incubated at 85 °C for 5 min. The following kit components were mixed together: cDNA Synthesis Premix, DTT and StarScript AMV Reverse Transcriptase. This reagent mix was added to the pre-heated RNA-mixtures and they were incubated at 25 °C for 5 min and then 42 °C for 20 min. Reactions were cooled down to 37 °C. Finishing solution was added to the samples and incubation was continued at 37 °C for an additional 10 min, then at 95 °C for 3 min. Samples were cooled down to 25 °C, and Terminal Tagging Premix, as well as DNA Polymerase were added. The incubation was continued at 25 °C for 15 min and then at 95 °C for 3 min. The di-tagged cDNA samples were purified by using the AMPure XP beads (Beckman Coulter). The purified samples were amplified by PCR using the FailSafe PCR Premix E, primers and FailSafe PCR enzyme (Lucigen, Epicentre). The PCR conditions were as follows: initial denaturation at 95 °C for 1 min, followed by 15 cycles at 95 °C for 30 sec 55 °C for 30 sec and 68 °C for 3 min. The final incubation step was carried out at 68 °C for 7 min. The PCR amplicons were purified with AMPure beads. The quantity and the quality of the samples were checked using Qubit fluorometer (Life Technologies) and Agilent 2100 Bioanalyzer, respectively.

### PacBio SMRTbell library preparation & sequencing – the non-amplified method & RSII sequencing

#### Generation of cDNAs

The SuperScript Double-Stranded cDNA Synthesis Kit (Life Technologies) was used to prepare cDNAs from the polyA(+) RNA samples. These samples were used to quantify the PRV transcriptome during the infection period between 1-12 h. The enzyme-which was included in the kit-was changed to SuperScript III Reverse Transcriptase. The first-strand synthesis reactions were primed with Anchored Oligo(dT)20 primers (Life Technologies, Table 5). The obtained cDNAs were measured by using Qubit HS dsDNA Assay Kit (Life Technologies). The total amount of cDNA synthesized at each time point was used to prepare SMRTbell templates.

### Preparation of SMRTbell libraries-“Barcoding method”

The cDNA samples (~500 ng/sample) were used to prepare SMRTbell templates by using the PacBio DNA Template Prep Kit 1.0 following the Pacific Biosciences’ 2 kb Template Preparation and Sequencing protocol.

#### Repairing the cDNA ends

Template Prep Buffer, ATP High, dNTP and End Repair Mix (PacBio) were added to the samples and then they were incubated at 25 °C for 15 min.

#### Sample purification 0.6x volume

AMPure PB bead was added to the samples. They were mixed using VWR vortex mixer for 10 min at 2000 rpm and room temperature. Tubes were placed in a magnetic bead rack for 3 min. After the bead pellets were formed, the supernatant were discarded. Beads were 2-times washed with freshly prepared ethanol (70%). Samples were dried and then they were eluted in 30 μl Elution Buffer (PacBio).

#### Adapter ligation

This step was carried out at 25 °C for 15 min with the addition of specific bar-coded adapters (Table 6), Template Prep Buffer, ATP Low and Ligase (PacBio). The enzyme was inactivated at 65 °C (10 min).

#### Exonuclease treatment

ExoIII (50U) and ExoVII (5U) enzymes were added to the carrier DNA-cDNA mixture, then they were incubated at 37 °C for 1 h, then the reactions were returned to 4 °C.

#### Sample purification

SMRTbell Templates were purified using 0.6× AMPure PB beads, as was described above. Two purification steps were applied after one other. The final elution volume was 10 μl. Qubit fluorometer was used for quantitation.

SMRTbell templates were bound to the PacBio’s P5 DNA polymerase. These complexes were bound to MagBeads using the Pacific Biosciences MagBead Binding Kit. The concentrations of the SMRTbell libraries were measured by Qubit and they were also qualified by Agilent 2100 Bioanalyzer. The PacBio RSII platform and C3 sequencing chemistry was used for sequencing. 180 min movies were applied for each SMRT Cell.

#### Annealing of the sequencing primers to the template DNA and the DNA polymerase binding

The PacBio Calculator v.2.3.0.0. was used to set the annealing and binding reactions. 2000 bp insert size and 1 ng/μl concentration was set. The Sequencing Primer (5000 nM) was diluted to 150 nM with the PacBio Elution Buffer (EB). One μl from the diluted primer and 10x Primer Buffer were added to the template DNA. Annealing was carried out at 80 °C for 2 min, and then the temperature was ramp to 25 °C at a rate of 0.1 °C/sec. The total volume of annealed template was bound to the Polymerase. For this, 2 μl dNTP, 2 μl DTT, 2 μl Binding Buffer (BB) and 2 μl diluted Polymerase were added to the samples. Mixtures were incubated at 30 °C for 4 h and then they were heated to 37 °C for 30 min. 2 μl from the complexes were used for MagBead binding. Cleaned MagBeads (74 μl) were added to the samples and they were incubated at 4 °C for 2 h on a HulaMixer rotator (Invitrogen). After the incubation, samples were washed with 19 μl BB, then 19 μl Wash Buffer (WB), and finally they were resuspended in 19 μl BB. The total amount of the MagBead-bound complex was loaded onto the RSII sequencer.

### Preparation of SMRTbell libraries-“Carrier DNA method”

The total amount of cDNA synthesized at each time point was used to prepare SMRTbell templates by using the PacBio DNA Template Prep Kit 1.0 and following the Pacific Biosciences template preparation and sequencing. protocol for Very Low (10 ng) Input 2 kb libraries with carrier DNA (pBR322, Thermo Scientific).

#### Preparing the carrier DNA

The concentration of the pBR322 plasmid DNA was measured by Nanodrop. A 100ng/μl stock solution was prepared from the plasmid using the PacBio Elution Buffer (EB). The DNA was exonuclease treated with the PacBio ExoIII (200U) and ExoVII (20U) enzymes and the Template Prep buffer (10×). The mixture was incubated at 37 °C for 1 h, then it was cooled down to 4 °C. The DNA was purified and concentrated by using 0.6× AMPure® PB beads and it was eluted in 50 μL EB. The exonuclease-treated carrier DNA was quantified by Qubit fluorometer.

#### Repairing the DNA damage

cDNA samples were mixed with DNA Damage Repair Buffer, NAD+, ATP High, dNTP and DNA Damage Repair Mix (all from the PacBio DNA Template Prep Kit), and then were incubated at 37 °C for 20 min. Samples were cooled to 4 °C.

#### Repairing the cDNA ends

End Repair Mix (PacBio) was added to the samples and then they were incubated at 25 °C for 5 min.

#### Sample purification 0.6x volume

AMPure PB bead was added to the samples and they were purified as in case of the barcoded samples.

#### Adapter ligation

This step was carried out at 25 °C for 60 min with the addition of Blunt Adapter, Template Prep Buffer, ATP Low and Ligase (PacBio). The enzyme was inactivated at 65 °C (10 min).

After this step, the ExoIII and ExoVII-treated carrier DNA (5 μl; 100 ng/μl) was mixed with the adapter-ligated cDNA samples (40 μl).

#### Exonuclease treatment

ExoIII (50U) and ExoVII (5U) enzymes were added to the carrier DNA-cDNA mixture, then they were incubated at 37 °C for 1 hour, then the reactions were returned to 4 °C.

#### Sample purification

Two purification steps were carried out successively, as was described earlier.

SMRTbell libraries were bound to DNA polymerases by using the DNA polymerase binding kit P5 and v2 sequencing primers. The DNA polymerase/template complexes were bound to MagBeads using the MagBead Binding Kit. The concentrations of the SMRTbell libraries were measured by Qubit and they were further analysed by Agilent 2100 Bioanalyzer. The cDNA sequencing reactions were carried out on the PacBio RSII platform with C3 sequencing chemistry with 180 min movies.

#### Annealing of the sequencing primers to the template DNA and the DNA polymerase binding

Conditioning and annealing of the Sequencing Primer, the binding of the Polymerase to the libraries, as well as Polymerase-template complex binding to the magnetic beads was done exactly as indicated by the PacBio Very Low Input protocol. The total amounts of prepared libraries (10 μl) were used for the binding. The DNA concentrations were set to 0.1 μl in the Calculator version 2.3.0.0. The “small-scale” preparation protocol and the “non-standard” protocol were chosen. The Sequencing Primer (5000 nM) was diluted to 150 nM in EB. One μl from the diluted primer and 10x Primer Buffer were added to the template DNA. Annealing was carried out at 80 °C for 2 min then the temperature was ramp to 25 °C at a rate of 0.1 °C/sec. The total volume of annealed template was bound to the Polymerase. For this, 2 μl dNTP, 2 μl DTT, 2 μl BB and 2 μl diluted Polymerase were added to the samples. Mixtures were incubated at 30 °C for 4 h and then they were heated to 37 °C for 30 min. The total volume from the polymerase binding step was used for MagBead binding. The salt molarity was adjusted for optimal binding by adding WB (0.3× volume) to the bound complex instead of BB. Cleaned MagBeads (26 μl) were added to the samples and they were incubated at 4 °C for 30 min on a HulaMixer rotator (Invitrogen). After the incubation, samples were washed with 26 μl BB, then 26 μl BW, and finally they were resuspended in 19 μl BB. The total amount of the MagBead-bound complex was loaded onto the PacBio machine.

### PacBio SMRTbell library preparation-Iso-Seq method/the amplified protocol & sequencing on RSII as well as Sequel platforms

Full-length cDNAs were generated using the Clontech SMARTer PCR cDNA Synthesis Kit based on the PacBio Isoform Sequencing (Iso-Seq) protocol. No Size Selection method was carried out for the analysis of short viral transcripts, while Manual Agarose-gel Size Selection, as well as SageELF™ and BluePippin™ Size-Selection Systems (Sage Science) were used for the isolation of long RNA molecules. The first-strand cDNAs were generated by using the SMARTer PCR cDNA Synthesis Kit (Clontech), the reactions were primed with oligo(dT) (part of the Clontech Kit) or adapter-linked GC-rich random primers (ordered from IDT DNA). The single-stranded cDNAs were PCR-amplified using KAPA HiFi Enzyme (Kapa Biosystems), in accordance with recommendations provided by PacBio, as follows: initial denaturation was carried at −95 °C for 2 min, followed by 16 cycles for PA-seq, 20 or 30 cycles for random-primed samples (the optimal cycle was determined in the optimization step) at −98 °C for 20 s (denaturation), −65 °C for 15 s (annealing) −72 °C for 4 min (extension). The final extension was carried out at −72 °C for 5 min. (n: 16 cycles was ideal for the No size-selection protocol. For the agarose size-selection, 12 cycles and 1:45 min extension was set for the amplification of transcripts between 2–3 kb and 15 cycles and 3 min extension was used for the longer transcripts. Sixteen cycles were set for the SageELF and BluePippin samples. PCR products were pooled then size selected manually by using 0.8% agarose gel or with the SageELF™ System according to the PacBio's protocol. Size-selected samples were amplified with KAPA enzyme using the conditions as above. The fraction of cDNAs with a size over 5 kb was run on BluePippin™ System to eliminate the short SMRTbell libraries. Five-hundred ng of each non-size-selected cDNA sample was applied for the SMRTbell template preparation, using the PacBio DNA Template Prep Kit 1.0. The amount of cDNAs from the size-selected samples used in the library preparation reaction were based on the following PacBio protocols: Procedure & Checklist – Isoform Sequencing (Iso-Seq™) using the Clontech SMARTer PCR cDNA Synthesis Kit and (a) Manual Agarose-gel Size Selection; (b) SageELF™ Size Selection System; and (c) BluePippin™ Size-Selection System. SMRTbell sequencing libraries were bound to polymerases by using the DNA/Polymerase Binding Kit P6 and v2 primers. The polymerase-template complexes were bound to MagBeads with the PacBio MagBead Binding Kit. The qualities of the samples were checked on the Agilent 2100 Bioanalyzer. Sequencing reactions were performed by using the PacBio RS II sequencer with DNA Sequencing Reagent 4.0. Movie lengths were 240 min or 360 min (one movie was recorded for each SMRT Cell).

The volume of the sequencing primer for the annealing, and the polymerase (P5 or P6) for the binding was determined using the PacBio Calculator version 2.3.1.1., by adding the concentrations and the average insert sizes of SMRTbell templates.

The polymerase-template complexes were bound to MagBeads, loaded onto SMRT Cells and sequenced on the RSII instrument.

The PacBio’s Binding Calculator was used to prepare the library for sequencing using the MagBead one-cell per well (OCPW) protocol, and binding kit P6v2 was used with an on-plate concentration of 0.05 nM. The insert sizes were set according to the size-selections which were applied: 1000, 2500 and 6000 bp sizes were chosen.

In short, the sequencing primer was diluted in PacBio EB to 150 nM. The annealing step was performed with 1 μl template DNA (cc: ~20 ng/μl), the diluted sequencing primer and primer buffer (10x). The final concentration of this mixture was 0.8333 nM. Annealing was carried out at 20 °C for 30 min then the DNA polymerase enzyme was diluted to a final concentration of 50 nM in PacBio BB v2, and then it was bound to the annealed template followed by the addition of DTT, dNTP and BB. The complex (0.5 nM final concentration) was incubated at 30 °C for 4 h. The sample complex (0.5 μl) was mixed with and 18.5 μl MagBead Binding Buffer (0.0125 nM final concentration). MagBeads were prepared as follows: 73.9 μl MagBeads were washed with 73.9 μl MagBead WB, then 73.9 μl MagBead BB was added. The sample complex was bound to the washed, prepared MagBeads for loading to the RSII machine: 19 μl sample complex was added to the beads, and then it was placed at 4 °C for 30 min in a HulaMixer. After incubation, the MagBead-bound complex was washed with 19 μl BB, then with 19 μl WB and finally, it was resuspended in 19 μl BB. The total amount of the MagBead-bound complex was loaded onto the instrument. The MagBead One Cell Per Well protocol was used. One SMRT Cell was also run on Sequel instrument.

#### Oxford Nanopore cDNA sequencing

PRV transcripts were sequenced on MinION device using the 1D Strand switching cDNA by ligation method (Version: SSE_9011_v108_revS_18Oct2016) and the ONT Ligation Sequencing Kit 1D (SQK-LSK108). For this, PolyA(+)-selected RNAs were used. 50ng from the samples were subjected to reverse transcription. Poly(T)-containing anchored primer [(VN)T_20_; ordered from Bio Basic, Canada, (Table 5)] and dNTPs (10 mM, Thermo Scientific) was added to the RNA samples and then the mixture was incubated at 65 °C for 5 min. Buffer and DTT from SuperScipt IV Reverse Transcriptase kit (Life Technologies), RNase OUT (Life Technologies) and strand-switching oligo with three O-methyl-guanine RNA bases (PCR_Sw_mod_3G; ordered from Bio Basic, Canada) were added and the sample was incubated at 42 °C for 2 min. 200U SuperScript IV Reverse Transcriptase enzyme was measured into the mix. First-strand cDNA synthesis was carried out at 50 °C for 10 min; it was followed by the strand switching step at 42 °C for 10 min. Enzymes were inactivated at 80 °C for 10 min. Five μl from the prepared double-stranded cDNA was amplified in a single PCR reaction using KAPA HiFi DNA Polymerase (Kapa Biosystems) and Ligation Sequencing Kit Primer Mix (provided by the 1D Kit). The Veriti Thermal Cycler (Applied Biosystems) was set as the 1D Kit’s protocol recommended: initial denaturation for 30 sec at 95 °C (1 cycle); denaturation for 15 sec at 95 °C (15 cycles); annealing for 15 sec at 62 °C (15 cycles); elongation for 4 min at 65 °C (15cycles); final extension 10 min at 65 °C. NEBNext End repair / dA-tailing Module (New England Biolabs) was used for end repair, while NEB Blunt/TA Ligase Master Mix (New England Biolabs) was applied for adapter ligations. The adapter sequences were supplied by the kit. Agencourt AMPure XP magnetic beads (Beckman Coulter) were used for purification following each enzymatic step. The Qubit Fluorometer (Life Technologies Qubit 2.0) and the Qubit (ds)DNA HS Assay Kit were used to quantify the concentration of the libraries. Samples were loaded on R9.4 SpotON Flow Cells, and base calling was performed using Albacore v1.2.6.

#### Oxford Nanopore sequencing on Cap-selected samples

To obtain full-length transcripts with the exact 5′-ends, Cap selection was carried out. For this, the TeloPrime Full-Length cDNA Amplification Kit (Lexogen) was used, which has an exceptional specificity for 5′-Cap. The starting material was 2 μg total RNA diluted in 12 μl water, from a mixed PRV sample (containing RNA from 1, 2, 3, 4, 5, 6, 7, 8, 12, 18 and 24 h post-infection). The method based on cDNA generation. Reverse transcription (RT) was carried out according to the kit’s manual. Briefly, the diluted RNA was mixed with RT buffer, primer (both are supplied by the kit). The RT primer contains an “oligodT” sequence (Table 5) to select the polyadenylated transcripts. The mixture was preheated at 70 °C for 30 sec, then it was cooled down to 37 °C for 1 min. RT enzyme and reagents (part of the kit) were added and the reaction was contain at 37 °C for 2 min. Temperature was increased to 46 °C for 50 min. The RNA-cDNA hybrid was purified using silica columns (kit’s component). A specific adapter was ligated to the cDNA by base-pairing of the 5’C to the cap structure of the RNA. This step was carried out by the double-strand specific ligase of the kit. Ligation was performed at 25 °C, overnight. The sample was purified after ligation using the silica columns. The cDNA was converted to dscDNA using the Second-Strand Mix and the Enzyme Mix from the Teloprime kit. The reaction was carried out in a Veriti Cycler with the following protocol: 98 °C for 90 sec, 62 °C for 60 sec, 72 °C for 5 min, hold at 25 °C.

Sample concentration was measured using Qubit dsDNA HS Assay Kit (Life Technologies). Specificity of the obtained cDNA was checked by qPCR (Rotor-Gene Q) using a gene specific primer (*us9*, 10μM each; Table 7), cDNA and ABsolute qPCR SYBR Green Mix (Thermo Fisher Scientific) in 20 μl final volume. The initial denaturation was 94 °C 15 min, and it was followed by 35 cycles of 94 °C for 25 sec, 60 °C 25 sec and 72 °C 6 sec.

The PolyA(+)-CAP-selected samples were also sequenced on MinION using the 1D Strand switching cDNA by ligation method. These samples were subjected to the end repair and adapter ligation steps, and then they were loaded on the ONT Flow Cells.

#### Oxford Nanopore direct RNA sequencing

Three flow cells were used for sequencing PRV samples following the Direct RNA sequencing (DRS) protocol from the ONT (Version: DRS_9026_v1_revM_15Dec2016). Total RNAs from 12 different time points were mixed together, and then polyA selection was carried out. RNA from the PolyA(+) fraction in 9 μl was used as a template for sequencing. RNA was mixed with the RT (oligodT-containing T_10_) adapter (supplied by the ONT Direct RNA Sequencing Kit; SQK-RNA001; Oxford Nanopore Technologies) and T4 DNA ligase (2M U/ml; New England BioLabs). The mixture was incubated at room temperature for 10 min. First-strand cDNA synthesis was carried out in 40 μl final volume with SuperScript III Reverse Transcriptase (Life Technologies), according to the DRS protocol, at 50 °C for 50 min, then 70 °C for 10 min in a Veriti Thermal Cycler. Samples were washed with Agencourt AMPure XP Beads (Beckman Coulter). XP Beads were treated before usage with RNase OUT (40 U/μl; Life Technologies); 2U enzyme was added to 1 μl bead. Purified RNA-cDNA hybrids were eluted in 20 μl Ambion Nuclease-Free Water (Thermo Fisher Scientific). RMX sequencing adapter was ligated to the eluted samples with T4 DNA ligase and NEBNext Quick Ligation Reaction Buffer (New England BiceoLabs) at room temperature for 10 min. Samples were purified with RNase OUT-treated XP beads using Wash Buffer (part of the DRS Kit) and then eluted in 21 μl Elution Buffer (provided by the DRS Kit). The concentration of the reverse-transcribed and adapted RNA was measured by using the Qubit 2.0 Fluorometer and Qubit dsDNA HS Assay Kit (Life Technologies). Samples were loaded onto the R9.4 SpotON Flow Cell.

Data on the quality of PacBio RSII, Sequel, and ONT MinION reads including insertions, deletions, and mismatches, as well as the coverages are summarized in Table 8 (available online only).

### Read processing

Raw reads from the random-primed Illumina sequencing were aligned to the PRV genome (KJ717942.1), using Tophat v2.09 (ref. 23); ambiguous reads were discarded. For PA-Seq, mapping was carried out with Bowtie v2 (ref. 24).

The PacBio RSII and Sequel consensus reads were generated following the RS_ReadsOfInsert protocol of the SMRT Analysis (v2.3.0 and v5.0.0) (Fig. 2), with the following settings: Minimum Full Passes=1, Minimum Predicted Accuracy=90, Minimum Length of Reads of Insert=1, Maximum Length of Reads of Insert=No Limit. These consensus reads were mapped using GMAP^25^, with the following settings: gmap -d Genome.fa --nofails -f samse File.fastq>Mapped_file.sam.

The ONT's Albacore software (v.2. 0.1) was used for base calling. This basecaller identify the nucleotide sequences directly from raw data. The sequencing reads were mapped with GMAP using the same setting as was described above.

Custom routines were used to acquire the quality information presented in this data descriptor. The codes have been archived on Github (https://doi.org/10.5281/zenodo.1034511).

## Data Records

All sequencing data have been uploaded to the European Nucleotide Archive under the project accession PRJEB24593 (Data Citation 1)-contains BAM files-and PRJEB9526 (Data Citation 2) – containing FASTQ files -. All sequencing reads were mapped to the KJ717942.1 genome build. All data can be used without restrictions.

## Technical Validation

The quantity of the isolated total RNAs, the polyA-selected RNAs, the rRNA-depleted samples, as well as the synthesized cDNA fractions and sequencing-ready libraries were measured by Qubit 2.0 (Life Technologies) fluorometer using the Qubit RNA, HS RNA and HS dsDNA Assay Kits. The conditions for primer annealing and binding of the polymerase to the templates were determined by PacBio’s Binding Calculator in RS Remote. The libraries were measured by Agilent 2100 Bioanalyzer using the Agilent High Sensitivity DNA Kit.

## Usage Notes

The provided dataset was primarily produced to discover and determine the complexity and expression dynamic properties of PRV transcriptome. The uploaded binary alignment (BAM) files contain reads already mapped to the KJ717942.1 reference. These aligned files can be further analysed using various bioinformatics program packages, such as bedtools^26^, samtools^27^, or visualized using e.g. IGV^28^, Geneious^29^ or Artemis^30^. The uploaded Illumina, PacBio and ONT files have not been trimmed, they contain terminal poly(A) sequences as well as the 5′and 3′ adapter sequences, which can be used to determine the orientations of the reads.

## Additional information

**How to cite this article**: Tombácz, D. *et al*. Transcriptome-wide survey of pseudorabies virus using next- and third-generation sequencing platforms. *Sci. Data* 5:180119 doi: 10.1038/sdata.2018.119 (2018).

**Publisher’s note**: Springer Nature remains neutral with regard to jurisdictional claims in published maps and institutional affiliations.

## Supplementary Material



## Figures and Tables

**Table 1:** Summary of the obtained read counts from long-read sequencing aligned to the PRV genome.

Sample^a^	Number of PRV reads^b^	Number of mapped reads^c^
MinION-Cap-selected	120394	131223
MinION-cDNA	44006	47363
MinION-RNA	29832	30667
Sequel	12555	13481
RSII-PolyA-amplified-1h	6956	6956
RSII-PolyA-amplified-4h	22958	22958
RSII-PolyA-amplified-8h	46147	46147
RSII-PolyA-amplified-1, 2, 4, 6, 8h-Bluepippin 0.8kb-5kb+	1077	1077
RSII-PolyA-amplified-1, 2, 4, 6, 8h-Bluepippin 0.8-2kb	7728	7728
RSII-PolyA-amplified-1, 2, 4, 6, 8h-Bluepippin 2-3kb	6131	6131
RSII-PolyA-amplified-1, 2, 4, 6, 8h-Bluepippin 3-5kb	3705	3705
RSII-PolyA-amplified-1, 2, 4, 6, 8h-Bluepippin 5kb+	4061	4061
RSII-PolyA-amplified-1, 4, 8h,-Manual Gel 1-2kb	3822	3822
RSII-PolyA-amplified-1h-Manual Gel 3kb+	3110	3110
RSII-PolyA-amplified-1h-Manual Gel 2-3kb	483	510
RSII-PolyA-amplified-4h-Manual Gel 3kb+	2645	2928
RSII-PolyA-amplified-4h-Manual Gel 2-3kb	14348	15708
RSII-PolyA-amplified-8h-Manual Gel 3kb+	3443	3765
RSII-PolyA-amplified-8h-Manual Gel 2-3kb	1608	1765
RSII-random-amplified-1, 2, 4, 6, 8h	1804	1953
RSII-random-amplified-1h	2327	2485
RSII-random-amplified-4h	7202	7629
RSII-random-amplified-8h	11452	12259
RSII-random-amplified-1, 4, 8h,-Manual Gel 3kb+	1778	1855
RSII-random-amplified-1, 4, 8h,-Manual Gel 1-2kb	2218	2425
RSII-random-amplified-1, 4, 8h,-Manual Gel 2-3kb	3393	3627
RSII-PolyA-non amplified-1h (1st)	74	81
RSII-PolyA-non amplified-1h (2nd)	7392	7440
RSII-PolyA-non amplified-2h (1st)	124	125
RSII-PolyA-non amplified-2h (2nd)	12944	13121
RSII-PolyA-non amplified-4h (1st)	1369	1574
RSII-PolyA-non amplified-4h (2nd)	1023	1054
RSII-PolyA-non amplified-6h (1st)	2144	2269
RSII-PolyA-non amplified-6h (2nd)	10151	10231
RSII-PolyA-non amplified-8h (1st)	219	237
RSII-PolyA-non amplified-8h (2nd)	7915	8015
RSII-PolyA-non amplified-12h (1st)	935	1002
RSII-PolyA-non amplified-12h (2nd)	12731	12864

^a^Type of the samples.

^b^Total number of PRV-specific reads.

^c^Total number of reads mapped to the PRV genome. (There is an approximately 15 kb-long inverted repeat sequence region in the PRV genome, therefore those reads which map to this location occur in duplicate in row **c**).

**Table 2:** Summary of the obtained read lengths from long-read sequencing.

Sample^a^	Avr. Read Length^b^	Avr. Read Length SD^c^
MinION-Cap-selected	810.00	519.07
MinION-cDNA	786.00	936.86
MinION-RNA	909.00	664.56
Sequel	1763.00	745.01
RSII-PolyA-amplified-1 h	1333.00	989.17
RSII-PolyA-amplified-4 h	1193.00	977.50
RSII-PolyA-amplified- 8h	1365.00	874.74
RSII-PolyA-amplified-1, 2, 4, 6, 8 h-Bluepippin 0.8kb-5 kb+	1555.00	938.80
RSII-PolyA-amplified-1, 2, 4, 6, 8 h-Bluepippin 0.8-2 kb	1362.00	381.91
RSII-PolyA-amplified-1, 2, 4, 6, 8 h-Bluepippin 2-3 kb	1300.00	474.58
RSII-PolyA-amplified-1, 2, 4, 6, 8 h-Bluepippin 3-5 kb	1048.00	489.94
RSII-PolyA-amplified-1, 2, 4, 6, 8 h-Bluepippin 5 kb+	1268.00	484.31
RSII-PolyA-amplified-1, 4, 8 h,-Manual Gel 1-2 kb	1251.00	532.61
RSII-PolyA-amplified-1 h-Manual Gel 3kb+	1805.00	1875.46
RSII-PolyA-amplified-1 h-Manual Gel 2-3 kb	1661.00	852.13
RSII-PolyA-amplified-4 h-Manual Gel 3kb+	2054.00	3654.40
RSII-PolyA-amplified-4 h-Manual Gel 2-3kb	1644.00	1874.46
RSII-PolyA-amplified-8 h-Manual Gel 3 kb+	1660.00	358.94
RSII-PolyA-amplified-8 h-Manual Gel 2-3 kb	1701.00	1162.41
RSII-random-amplified-1, 2, 4, 6, 8 h	772.00	382.48
RSII-random-amplified-1 h	1121.00	438.93
RSII-random-amplified-4 h	1109.00	557.74
RSII-random-amplified-8 h	1105.00	468.34
RSII-random-amplified-1, 4, 8 h,-Manual Gel 3 kb+	1726.00	2107.78
RSII-random-amplified-1, 4, 8 h,-Manual Gel 1-2 kb	999.00	522.86
RSII-random-amplified-1, 4, 8 h,-Manual Gel 2-3 kb	1173.00	1725.91
RSII-PolyA-non amplified-1 h (1st)	1077.00	323.67
RSII-PolyA-non amplified-1 h (2nd)	1403.00	625.65
RSII-PolyA-non amplified-2 h (1st)	1227.00	428.39
RSII-PolyA-non amplified-2 h (2nd)	1331.00	608.73
RSII-PolyA-non amplified-4 h (1st)	1207.00	529.63
RSII-PolyA-non amplified-4 h (2nd)	1307.00	585.04
RSII-PolyA-non amplified-6 h (1st)	1189.00	485.97
RSII-PolyA-non amplified-6 h (2nd)	1211.00	473.44
RSII-PolyA-non amplified-8 h (1st)	1120.00	443.99
RSII-PolyA-non amplified-8 h (2nd)	1390.00	554.59
RSII-PolyA-non amplified-12 h (1st)	1081.00	410.88
RSII-PolyA-non amplified-12 h (2nd)	1336.00	555.02

^a^Type of the samples.

^b^Average read lengths of the different library preparation and long-read sequencing approaches.

^c^Standard deviation (SD) values.

**Table 3:** Summary table of the various wet lab approaches used in this study.

Sample No	Sample	Sample time points (h pi)	RT priming	Cap-selection	Amplification	Size selection	Library prep	Platform
1	Mixed	1, 2, 4, 6, 8, 10, 12, 14, 16, 18, 20, 22, 24	Oligo(d)T	no	yes	no	Illumina single-end	HiScan SQ
2	Mixed	1, 2, 4, 6, 8, 10, 12, 14, 16, 18, 20, 22, 24	Random	no	yes	no	Illumina paired-end	HiScan SQ
3	1 h	1	Oligo(d)T	no	yes	no	PacBio Isoform seq	RSII
4	4 h	4	Oligo(d)T	no	yes	no	PacBio Isoform seq	RSII
5	8 h	8	Oligo(d)T	no	yes	no	PacBio Isoform seq	RSII
6	Mixed	1, 2, 4, 6, 8	Oligo(d)T	no	yes	Bluepippin 5kb+	PacBio Isoform seq	RSII
7	Mixed	1, 2, 4, 6, 8	Oligo(d)T	no	yes	Bluepippin 0.8-2kb	PacBio Isoform seq	RSII
8	Mixed	1, 2, 4, 6, 8	Oligo(d)T	no	yes	Bluepippin 2-3kb	PacBio Isoform seq	RSII
9	Mixed	1, 2, 4, 6, 8	Oligo(d)T	no	yes	Bluepippin 3-5kb	PacBio Isoform seq	RSII
10	Mixed	1, 2, 4, 6, 8	Oligo(d)T	no	yes	Bluepippin 5kb+	PacBio Isoform seq	RSII
11	Mixed	1, 4, 8	Oligo(d)T	no	yes	Manual Gel 1-2kb	PacBio Isoform seq	RSII
12	1 h	1	Oligo(d)T	no	yes	Manual Gel 3kb+	PacBio Isoform seq	RSII
13	1 h	1	Oligo(d)T	no	yes	Manual Gel 2-3kb	PacBio Isoform seq	RSII
14	4 h	4	Oligo(d)T	no	yes	Manual Gel 3kb+	PacBio Isoform seq	RSII
15	4 h	4	Oligo(d)T	no	yes	Manual Gel 2-3kb	PacBio Isoform seq	RSII
16	8 h	8	Oligo(d)T	no	yes	Manual Gel 3kb+	PacBio Isoform seq	RSII
17	8 h	8	Oligo(d)T	no	yes	Manual Gel 2-3kb	PacBio Isoform seq	RSII
18	Mixed	1, 2, 4, 6, 8	Random	no	yes	no	PacBio Isoform seq	RSII
19	1 h	1	Random	no	yes	no	PacBio Isoform seq	RSII
20	4 h	4	Random	no	yes	no	PacBio Isoform seq	RSII
21	8 h	8	Random	no	yes	no	PacBio Isoform seq	RSII
22	Mixed	1, 4, 8	Random	no	yes	Manual Gel 3kb+	PacBio Isoform seq	RSII
23	Mixed	1, 4, 8	Random	no	yes	Manual Gel 1-2kb	PacBio Isoform seq	RSII
24	Mixed	1, 4, 8	Random	no	yes	Manual Gel 2-3kb	PacBio Isoform seq	RSII
25	1 h	1	Oligo(d)T	no	no	no	PacBio 2kb	RSII
26	1 h	1	Oligo(d)T	no	no	no	PacBio Very Low Input	RSII
27	2 h	2	Oligo(d)T	no	no	no	PacBio 2kb	RSII
28	2 h	2	Oligo(d)T	no	no	no	PacBio Very Low Input	RSII
29	4 h	4	Oligo(d)T	no	no	no	PacBio 2kb	RSII
30	4 h	4	Oligo(d)T	no	no	no	PacBio Very Low Input	RSII
31	6 h	6	Oligo(d)T	no	no	no	PacBio 2kb	RSII
32	6 h	6	Oligo(d)T	no	no	no	PacBio Very Low Input	RSII
33	8 h	8	Oligo(d)T	no	no	no	PacBio 2kb	RSII
34	8 h	8	Oligo(d)T	no	no	no	PacBio Very Low Input	RSII
35	12 h	12	Oligo(d)T	no	no	no	PacBio 2kb	RSII
36	12 h	12	Oligo(d)T	no	no	no	PacBio Very Low Input	RSII
37	Mixed	1, 2, 4, 6, 8	Oligo(d)T	no	yes	no	PacBio Isoform seq	Sequel
38	Mixed	1, 2, 3, 4, 5, 6, 7, 8, 9, 12, 18, 24	Oligo(d)T	no	no	no	ONT Direct RNA	MinION
39	Mixed	1, 2, 3, 4, 5, 6, 7, 8, 9, 12, 18, 24	Oligo(d)T	yes	yes	no	ONT cDNA	MinION
40	Mixed	1, 2, 3, 4, 5, 6, 7, 8, 9, 12, 18, 24	Oligo(d)T	no	yes	no	ONT cDNA	MinION

**Table 4:** Summary table of the reagents and chemistries used for the sequencing.

Total RNA isolation	PolyA selection	Ribodepletion	Reverse transcription & dscDNA production	cDNA synthesis by PCR	Library preparation kit	Sequencing chemistry	Instrument
Macherey-Nagel RNA	Qiagen Oligotex mRNA mini Kit	–	StarScript AMV Reverse Transcriptase	FailSafe PCR PreMix Selection Kit	ScriptSeq v2 RNA-Seq Library Preparation Kit	NA	HiScan 2500
	–	Epicentre Ribo-Zero™ Magnetic Kit H/M/R					
	Qiagen Oligotex mRNA mini Kit	–	SuperScript III & SuperScript double-stranded cDNA Synthesis Kit	–	PacBio Template Preparation Kit	P5-C3	RSII
			Clontech SMARTer PCR cDNA Synthesis Kit	KAPA HiFi PCR Kit		P6-C4	
	–	Epicentre Ribo-Zero™ Magnetic Kit H/M/R					
	Qiagen Oligotex mRNA mini Kit	–					Sequel
			SuperScript III	–	Direct RNA Sequencing Kit	NA	MinION
			SuperScript IV	KAPA HiFi PCR Kit	Ligation Sequencing Kit 1D	NA	
			Lexogen Teloprime Kit enzymes & reagents	Lexogen Teloprime PCR mix	Ligation Sequencing Kit 1D	NA	

**Table 5:** The list of primers used in this study for the reverse transcription reactions.

Sequencing method	Name, availability	Catalog #	Sequence (5' -> 3')
Illumina PolyA	Custom made-adaptor-primer VN(T)_20_ (IDT DNA)	-	GTGTGCTCTTCCGATCT(T)_20_VN
Illumina random	Random hexamer + tagging sequence-ScriptSeq™ v2 RNA-Seq Library Preparation Kit (Epicentre)	SSV21106 & SSV21124	GTGTGCTCTTCCGATCTNNNNNN
PacBio non-amplified	Anchored Oligo(dT)20 primers (Life Technologies)	12577011	TTTTTTTTTTTTTTTTTTTTVN
PacBio amplified PolyA	3' SMART CDS primer II A-SMARTer PCR cDNA Synthesis Kit (Clontech)	634925 & 634926	AAGCAGTGGTATCAACGCAGAGTAC(T)_30_VN
PacBio amplified Random	Custome made (IDT DNA)	-	AAGCAGTGGTATCAACGCAGAGTACNNNNNN (G: 37%; C: 37%; A: 13%; T: 13%)
MinION cDNA	Poly(T)-containing anchored primer [(VN)T20-ONT recommended, custom made (Bio Basic)	-	5phos/ ACTTGCCTGTCGCTCTATCTTC(T)_20_VN
MinION CAP selected	TeloPrime Full-Length cDNA Amplification Kit (Lexogen)	013.08 & 013.24	TCTCAGGCGTTTTTTTTTTTTTTTTTT
MInION RNA	RT adapter-Direct RNA Sequencing Kit (Oxford Nanopore Technologies)	SQK-RNA001	GAGGCGAGCGGTCAATTTTCCTAAGAGCAAGAAGAAGCCTTTTTTTTTT

**Table 6:** Barcode sequences utilized for PacBio sequencing.

Name	Sequence
PBBC adapter #1	[Phos] TATGCTAATCTCTCTCTTTTCCTCCTCCTCCGTTGTTGTTGTTGAGAGAGATTAGCATA
PBBC adapter #2	[Phos] GACAGTGATCTCTCTCTTTTCCTCCTCCTCCGTTGTTGTTGTTGAGAGAGATCACTGTC
PBBC adapter #3	[Phos] GATCTCGATCTCTCTCTTTTCCTCCTCCTCCGTTGTTGTTGTTGAGAGAGATCGAGATC
PBBC adapter #4	[Phos] TACACGTATCTCTCTCTTTTCCTCCTCCTCCGTTGTTGTTGTTGAGAGAGATACGTGTA
PBBC adapter #5	[Phos] GAGCTCAATCTCTCTCTTTTCCTCCTCCTCCGTTGTTGTTGTTGAGAGAGATTGAGCTC
PBBC adapter #6	[Phos] TCTGCAGATCTCTCTCTTTTCCTCCTCCTCCGTTGTTGTTGTTGAGAGAGATCTGCAGA
This table contains the modified adapter sequences with unique barcodes which were used for multiplex sequencing. The barcode sequences are labelled by blue colour.	

**Table 7:** The gene-specific primers used for the amplification of *us9* gene of PRV.

Primer	Sequence
Forward	CAGGACGACTCGGACTGCTA
Reverse	AGGAACTCGCTGGGCGT

**Table 8:** Summary table of the read qualities obtained from PacBio and ONT long-read sequencing

Samplifiedle^a^	Del %^b^	Del SD^c^	Ins %^d^	Ins SD^e^	MM %^f^	MM SD^g^	Coverage^h^
Illumina-randomom	**0.01**	0.09	**0.01**	0.11	**0.45**	0.64	**317.86**
Illumina-PolyA	**0.01**	0.14	**0.01**	0.19	**0.33**	0.94	**3095.39**
RSII-PolyA-Seq-amplified- 1 h	**1.24**	1.73	**1.24**	3.05	**0.82**	1.90	**54.82**
RSII-PolyA-Seq-amplified-4 h	**1.96**	2.40	**1.03**	2.72	**0.83**	1.84	**160.97**
RSII-PolyA-Seq-amplified-8 h	**1.72**	1.95	**1.13**	2.88	**0.86**	1.89	**378.37**
RSII-PolyA-Seq-amplified-1, 2, 4, 6, 8 h-BluePippin 0.8 kb-5 kb+	**0.76**	1.43	**0.45**	2.61	**1.14**	3.89	**9.07**
RSII-PolyA-Seq-amplified-1, 2, 4, 6, 8 h-BluePippin 0.8-2 kb	**0.53**	0.98	**0.05**	0.37	**0.06**	0.17	**67.26**
RSII-PolyA-Seq-amplified-1, 2, 4, 6, 8 h-BluePippin 2-3 kb	**0.39**	1.01	**0.06**	0.31	**0.06**	0.19	**50.15**
RSII-PolyA-Seq-amplified-1, 2, 4, 6, 8 h-BluePippin 3–5 kb	**0.51**	1.17	**0.07**	0.40	**0.07**	0.18	**23.03**
RSII-PolyA-Seq-amplified-1, 2, 4, 6, 8 h-BluePippin 5 kb+	**0.39**	0.95	**0.09**	0.78	**0.06**	0.21	**32.45**
RSII-PolyA-Seq-amplified-1, 4, 8 h,-Manual gel size selection 1-2 kb	**0.83**	1.46	**0.06**	0.74	**0.07**	0.24	**29.85**
RSII-PolyA-Seq-amplified-1 h-Manual gel size selection 3 kb+	**1.62**	2.10	**0.86**	2.35	**0.83**	2.06	**32.43**
RSII-PolyA-Seq-amplified-1 h-Manual gel size selection 2–3 kb	**0.96**	1.37	**0.69**	2.63	**0.49**	1.56	**4.75**
RSII-PolyA-Seq-amplified-4 h-Manual gel size selection 3 kb+	**1.56**	1.95	**0.91**	2.99	**0.77**	2.09	**28.08**
RSII-PolyA-Seq-amplified-4 h-Manual gel size selection 2–3 kb	**1.49**	1.91	**0.92**	2.61	**0.79**	2.02	**134.18**
RSII-PolyA-Seq-amplified-8 h-Manual gel size selection 3 kb+	**0.91**	1.43	**0.05**	0.37	**0.06**	0.15	**37.50**
RSII-PolyA-Seq-amplified-8 h-Manual gel size selection 2–3 kb	**1.56**	1.82	**0.76**	2.38	**0.63**	1.73	**16.89**
RSII-random-amplified-1, 2, 4, 6, 8 h	**0.57**	1.32	**0.17**	1.02	**0.13**	0.63	**8.26**
RSII-random-amplified-1 h	**0.78**	1.44	**0.10**	0.98	**0.09**	0.46	**16.11**
RSII-random-amplified-4 h	**0.80**	1.46	**0.10**	0.75	**0.09**	0.37	**46.73**
RSII-random-amplified-8 h	**0.68**	1.33	**0.11**	0.97	**0.09**	0.38	**75.37**
RSII-random-amplified-1, 4, 8 h,-Manual gel size selection 3 kb+	**1.05**	1.51	**1.02**	2.84	**0.73**	1.93	**16.73**
RSII-random-amplified-1, 4, 8 h,-Manual gel size selection 1–2 kb	**0.72**	1.37	**0.15**	1.03	**0.10**	0.42	**11.61**
RSII-random-amplified-1, 4, 8 h,-Manual gel size selection 2–3 kb	**0.90**	1.45	**0.64**	2.24	**0.45**	1.42	**22.93**
RSII-PolyA-Seq-non amplified-1 h (Barcoding method)	**0.55**	0.97	**0.51**	0.92	**0.24**	0.51	**0.53**
RSII-PolyA-Seq-non amplified-1 h (Carrier DNA method)	**0.63**	1.05	**0.92**	1.56	**0.31**	0.70	**70.25**
RSII-PolyA-Seq-non amplified-2 h (Barcoding method)	**0.49**	0.65	**0.50**	0.79	**0.17**	0.43	**1.02**
RSII-PolyA-Seq-non amplified-2 h (Carrier DNA method)	**0.47**	0.85	**1.07**	1.77	**0.30**	0.60	**115.89**
RSII-PolyA-Seq-non amplified-4 h (Barcoding method)	**0.43**	0.75	**0.46**	0.96	**0.16**	0.47	**10.81**
RSII-PolyA-Seq-non amplified-4 h (Carrier DNA method)	**0.75**	1.17	**0.94**	1.56	**0.41**	0.72	**8.96**
RSII-PolyA-Seq-non amplified-6 h (Barcoding method)	**0.50**	0.84	**0.47**	1.10	**0.17**	0.39	**16.76**
RSII-PolyA-Seq-non amplified-6 h (Carrier DNA method)	**0.74**	1.07	**0.98**	1.49	**0.41**	0.72	**82.06**
RSII-PolyA-Seq-non amplified-8 h (Barcoding method)	**0.42**	1.02	**0.34**	0.70	**0.11**	0.28	**1.62**
RSII-PolyA-Seq-non amplified-8h (Carrier DNA method)	**0.75**	0.98	**1.03**	1.65	**0.44**	0.70	**74.77**
RSII-PolyA-Seq-non amplified-12h (Barcoding method)	**0.44**	0.74	**0.35**	0.74	**0.16**	0.40	**6.67**
RSII-PolyA-Seq-non amplified-12 h (Carrier DNA method)	**0.65**	0.86	**1.58**	2.10	**0.52**	0.77	**114.10**
Sequel	**0.13**	0.48	**0.46**	1.42	**0.15**	0.55	**136.75**
MinION-Cap-selected	**5.77**	1.96	**4.11**	4.29	**7.12**	2.48	**328.53**
MinION-cDNA	**6.43**	2.31	**3.22**	2.28	**8.14**	2.80	**151.94**
MinION-RNA	**8.68**	2.63	**2.56**	2.14	**7.81**	2.24	**162.42**
Data show that the ONT MinION sequencing resulted in relatively high error rates for both Indels and mismatches. The best read quality (i.e. less Indel) – as expected from the literature data – was obtained from the Illumina runs; however, we obtained no significant differences between the Illumina and PacBio mismatch data. Moreover, several PacBio data sets yielded better results than those of the Illumina assemblies. The composition of the errors of the three platforms (Illumina, PacBio, and ONT) and the four techniques (HiScan SQ, RSII, Sequel, and MinION) are different. Mismatches are the most common errors in both ONT (according to the previously published data^31^) and Illumina data^32^. In agreement with others’ data^31^, insertions are the least frequent errors in ONT in our study. However, in contrast to the others’ results^31^, insertions are the major errors in our PacBio RSII and Sequel data. In sum, the absolute error rate of both the Illumina and PacBio (especially the Sequel) platforms is fairly low. The custom code [Github (https://doi.org/10.5281/zenodo.1034511)] was used to obtain the presented data. In Illumina sequencing experiments, the non-matching nucleotides, generated by the mapping software, were removed from the end of alignments in SAM files in order to get more precise quality values.							

^a^Samples

^b^percentage of deletions.

^c^standard deviations.

^d^percentage of insertions.

^e^standard deviations.

^f^percentage of mismatches.

^g^standard deviations of mismatches.

^h^average coverages across the PRV genome.

## References

[d1] European Nucleotide Archive 2018 PRJEB24593

[d2] European Nucleotide Archive 2015 PRJEB9526

